# A Review on Leaching of Spent Lithium Battery Cathode Materials Adopting Deep Eutectic Solvents

**DOI:** 10.1002/open.202400258

**Published:** 2024-12-06

**Authors:** Chongyu Li, Jianjiao Jin, Zhang Yuan, Chenyun Zhang, Liangqin Wu, Chu Wang

**Affiliations:** ^1^ Shazhou Professional Institute of Technology No.1 FuXin Road Zhangjiagang 215600 PR China; ^2^ Wuxi Vocational Institute of Arts & Technology Yixing 214206 China

**Keywords:** Spent lithium-ion batteries, Deep eutectic solvent, Leaching of positive electrode materials, Acidity, Reducibility, Coordination

## Abstract

As a result of the swift surge in the adoption of electric vehicles, the quantity of spent lithium‐ion power batteries has been growing at an exponential rate. Improper handling of these batteries can lead to the waste of strategic metal resources and pose risks to the environment and human health. Without doubt, it is essential to scientifically recover and reuse these spent power batteries, particularly by recovering positive electrode materials. Currently, there are several methods for recovering positive electrode materials, including pyrometallurgy, hydrometallurgy, bioleaching, and deep eutectic solvents (DESs) leaching. This review concetrated on the emerging technology of DESs leaching for positive electrode materials in spent lithium‐ion battery. It provided an overview of the latest advancements in DESs leaching, considering factors such as acidity, reducibility, and coordination of DESs. The current technical status was analyzed and discussed, while also addressing the challenges and prospects for the development of DESs recovery in spent Li‐ion power batteries. This work aims to offer practical guidance and serve as a foundation for additional studies and widespread implementation of DESs leaching for positive electrode materials.

## Graphical abstract

The leaching and recovery of spent lithium batteries (SLiB) using deep eutectic solvents (DESs) have received widespread attention. This review summarizes the latest advancements in the leaching of SLiB cathode materials with DESs in terms of acidity, reducibility, and coordination. It analyzes the current technical status and explores the development challenges and prospects of DESs in the recycling of SLiB.

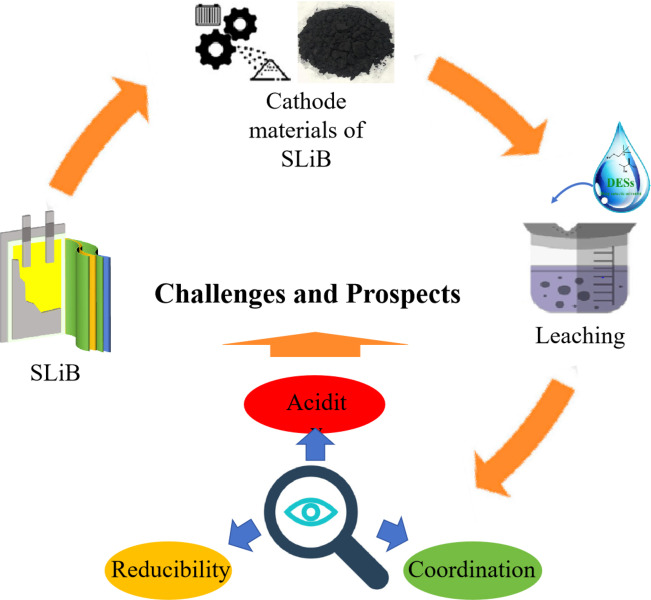

## 1 Introduction

**Figure 1:**
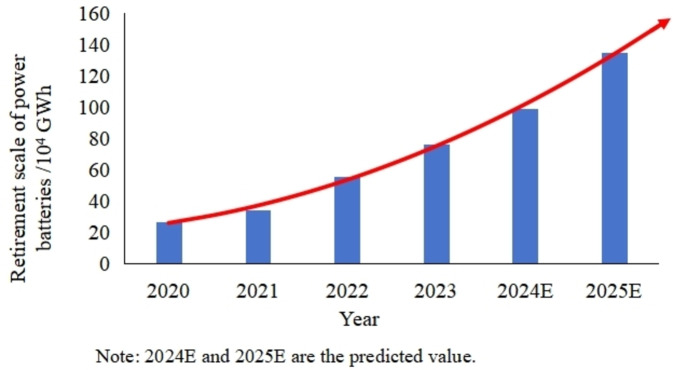
Trend of spent power battery scale.

With the introduction of carbon neutrality targets, the electric vehicle industry is nudergoing sharply development. However, it will also face a massive wave of spent Li‐ion power batteries (as shown in Figure 1). It is estimated that the volume of spent power batteries will achieve 1.35 million GWh in China by 2025.^[1]^

**Figure 2:**
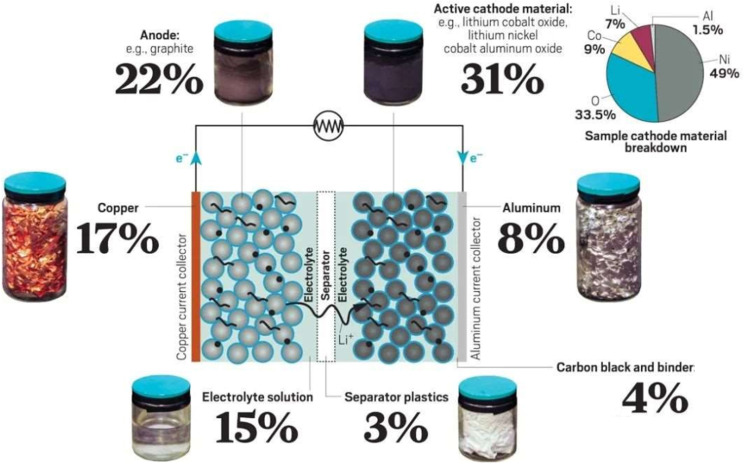
Materials composition of ternary Li‐ion batteries.^[3]^

Improper handling of scrapped lithium‐ion batteries will lead to serious problems: (1) Cobalt, nickel, manganese, and electrolytes in power batteries can easily leak from the casing, polluting soil and groundwater, posing a threat to the environment and public health; (2) It creates a security issue for scarce resources. Lithium, cobalt, nickel, etc., are all scarce resources in China and are heavily dependent on imports. Waste batteries are similar to high‐purity metal ore deposits,[^2^, ^3^] as shown in Figure 2. Therefore, the effective recycling of high‐quality materials in Li‐ion batteries can both reduce environmental pollution and alleviate the scarcity of metals like Li, Co, Mn, and Ni. Obviously, the recycling of cathode materials in spent power batteries is necessary and urgent.

To date, countries around the world are actively researching recycling methods for spent Li‐ion power batteries, as shown in Table 1, 2.^[4]^ Currently, existing recycling processes mainly utilize hydrometallurgical and pyrometallurgical techniques to recover valuable metals (including Co, Ni, Li, etc.) in cathode materials. Additionally, there are also some approaches that employ biohydrometallurgical techniques for the recycling of spent Li‐ion batteries.

**Table 1:** Typical recycling methods employed by various commercial entities.

Organization	Implementation Stage	Country	Technology solution	Recycling scale (ton)
Akkuser Oy	Commercial	Finland	Mechanical treatment	1000
Umicore	Commercial	Belgium	High temperature/wet smelting	7000
Li‐cycle	Test	Canada	Mechanical treatment	7500
Retriev Technologies	Test	USA	Mechanical treatment	4500
Accurec	Commercial	Germany	Mechanical treatment and Pyrometallurgical smelting	2500
Valdi	Commercial	France	Mechanical treatment and wet smelting	20000
JX Nippon Mining and Metals	Commercial	Japan	High temperature/wet smelting	5000
GEM	Commercial	China	Mechanical treatment and wet smelting	10000
Brunp recycling	Commercial	China	Mechanical treatment and wet smelting	25000–30000

**Table 2:** Leaching Effect of Partial DESs.

DESs	S/L	Conditon		Leaching efficiency	References
		T / °C	Time /h		
2ChCl : LA		70	24	Li, Ni, Co, Mn=100 %	[53]
ChCl : MAL : PTSA	20 g/L	100	24	Co : 98.61; Li : 98.78 %	[56]
Chcl : 2FA+10 % H₂O	1 g/50 g	70	0.17	Co, Li=100 %	[58]
Chcl : OA	20 mg/g	120	2	Li, Mn>96 %	[60]
CHCl : OA : 2H_2_0	20 g/L	100	0.17	Li : 99.05 %; Co : 99.21 %	[61]
Chcl : OA : 8H₂O +Microwave	1 g/50 mL	100	0.17	Li : 99.05 %; Co : 99.21 %	[61]
Chcl : PTSA : H₂O	0.1 g/g	90	0.25	Co, Li=100 %	[62]
ChCl : BSA : 2EA	20 g/L	90	2	Co : 98 %; Li : 99 %	[71]
ChCl : EG	12.5 g/L	180	240	Li : 91.63 %; Co : 92.52; Ni : 94.92 %; Mn : 95.47 %	[77]
2ChCl : CA+35 %H_2_O	20 g/L	40	1	Co>98 %	[90]
ChCl : 2Gly	2.5 g/L	180	2	Co : 47.59 %; Li : 50.4 %	[116]
2ChCl : 1.6LAA	1 g/25 g	50	1	Ni, Co, Mn>96 %	[116]
ChCl : Urea : 2EG	9.69 g/L	100	72	Co : 1.61 %; Li : 92.82	[117]

**Figure 3:**
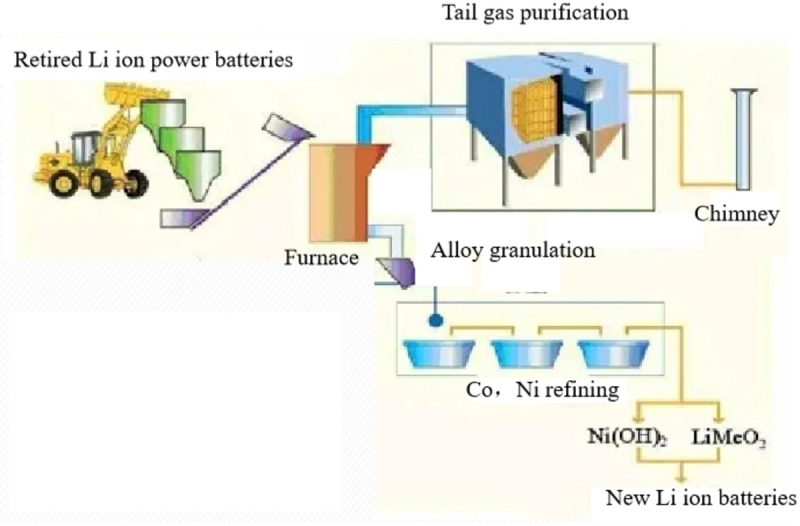
Process of pyrometallurgical technique.

The pyrometallurgical process, as shown in Figure 3, typically involves high‐temperature smelting in a furnace. It has the advantages of a simple and convenient processing technique, making it suitable for handling batteries with complex compositions. However, it also has significant drawbacks, such as high energy consumption, stringent equipment requirements, high costs, and high temperatures (above 1400 °C).[^5^, ^6^, ^7^] Additionally, this process often generates various harmful gases, causing secondary pollution to the atmosphere,^[8]^ and the recycling efficiency is not ideal.

**Figure 4:**
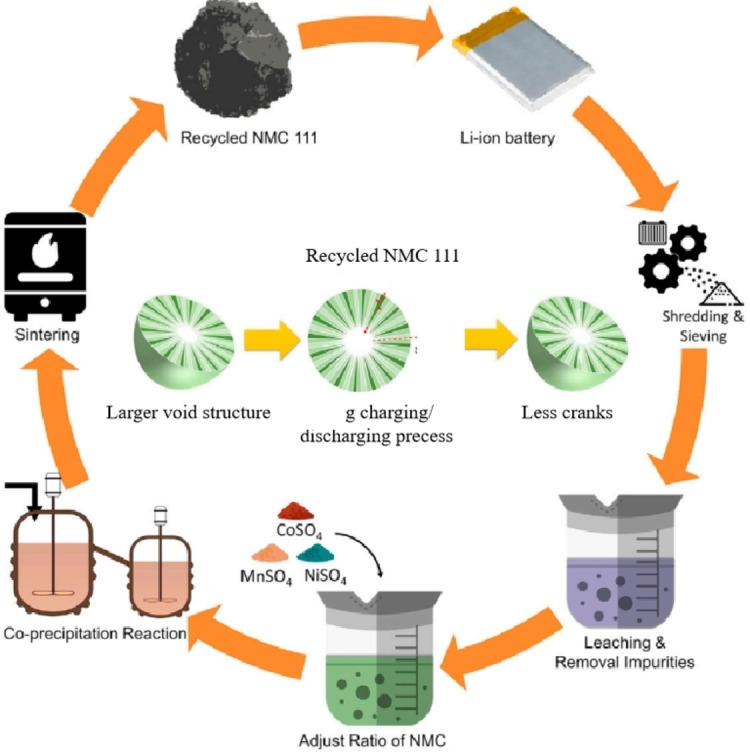
Wet Metallurgical Process Flow.

The wet metallurgical process, as shown in Figure 4, involves using appropriate chemical reagents to leach the cathode material, allowing the elements (Li, Co, Ni, Mn, etc.) transfer into the liquid phase for subsequent separation and recovery or wet regeneration.^[9]^ This process is currently the main method of recycling because of its relatively better recovery efficiency and the ease of achieving continuous and automated production.

The Conventional hydrometallurgical processes primarily employ inorganic acids, including hydrochloric acid,[^10^, ^11^, ^12^] sulfuric acid,[^13^, ^14^, ^15^, ^16^, ^17^, ^18^] nitric acid,^[11]^ and phosphoric acid.[^19^, ^20^, ^21^, ^22^] However, during the leaching process with inorganic strong acids, toxic gases like chlorine and sulfur trioxide are often generated. Therefore, conventional wet metallurgical methods have serious environmental issues, mainly becuase of the large amounts of consumption for chemical reagents, long reaction times, and the generation of secondary pollutants.^[23]^ They often involve cumbersome procedures for harmless treatment, which are harmful to both workers and the environment. For example, the commonly used hydrochloric acid[^24^, ^25^, ^26^] with hydrogen peroxide system in wet metallurgy needs massive acid and generates a significant amount of chemical waste. The excess residual acid and chlorine gas from the excessive reaction both contribute to pollution. The same problems occur in sulfuric acid systems, where sulfur dioxide and residual acid also pose similar issues. Additionally, the difficulty and high cost of separating closely related metal elements further exacerbate the challenges. Therefore, the current recycling technologies suffer from severe pollution and low efficiency, contradicting the concept of a carbon‐neutral economy. Currently, comprehensive reviews on wet metallurgical methods mainly focus on the research of different leaching agents and reducing agents in conventional acid leaching methods for the recovery of valuable metals in spent power batteries. Although traditional acid leaching methods have high leaching efficiency, they are highly polluting, hazardous, and costly.^[27]^ Hence, organic acids have been studied as alternatives to hazardous inorganic acids.[^28^, ^29^, ^30^] Currently, commonly researched organic acids for this purpose include formic acid (FA),^[31]^ oxalic acid (OA),^[11]^ citric acid (CA),[^32^, ^33^, ^34^] benzenesulfonic acid,^[35]^ succinic acid,^[36]^ acetic acid(AA), gluconic acid,^[37]^ Benzoic acid (BA), malic acid (MAL),^[29]^ p‐toluenesulfonic acid (PTSA) and lactic acid (LA), etc. Nevertheless, the high‐temperature stability and toxicity of organic acids are important limitations to their development.^[38]^

Compared to traditional pyrometallurgy and hydrometallurgy, bioleaching offers mild leaching conditions, an environmentally friendly system with minimal pollution, no generation of harmful gases, and minimal consumption of reagents. It also requires less sophisticated equipment and incurs lower associated costs. However, the bioleaching system is not yet mature compared to pyrometallurgy and hydrometallurgy. The adaptability of cultivated strains is poor, and the cultivation cycle is long, which severely limits the industrial application of bioleaching. If specific strains with short cultivation cycles and high efficiency in adsorbing metal ions can be cultivated, bioleaching will become an ideal approach for recovering spent Li‐ion batteries.^[39]^

Therefore, it is critical to explore a solvent that is eco‐friendly, low in volatility, low in cost, and capable of leaching metal oxides. In recent years, the discovery of deep eutectic solvent(DES) has provided a new approach for the recovering of spent Li‐ion batteries.

DES refers to a new type of green solvent, which is a mixture of hydrogen bond acceptors (usually quaternary ammonium salts) and hydrogen bond donors (usually alcohols, acids, or amides) in a certain proportion. The melting point of the mixture is significantly lower than that of each raw material (which can be higher than −73.15 °C).^[40]^ The sharp decrease in melting point is due to the large ionic asymmetry and the low lattice energy, which is beneficial to the liquid state. Hydrogen bonds between asymmetric ions (such as halogen ions) in the hydrogen bond acceptor and the hydrogen bond donor interact to generate charge delocalization,^[41]^ while larger asymmetric ions will reduce the lattice energy of the mixture.^[42]^ Currently, DESs based on choline chloride (HOCH_2_CH_2_N(CH_3_)^3+^Cl^−^, ChCl) are the most common, including ChCl/ethylene glycol (EG), ChCl/glycerol, ChCl/urea, etc. DESs exhibit unexpected dissolution capabilities for various metal compounds. The existence of coordinating ligands in DES plays a key role in dissolving metal ions, and the proton with acidic properties acting as an oxygen acceptor also contributes to the dissolution. The dissolution of metal oxides is a key process in a series of important processes including metal purification and electroplating.^[42]^ Since the concept was first reported by Abbott et al. in 2003,^[40]^ it has attracted attention from various fields such as chemistry, materials science, and environmental science, and has been widely applied in numerous areas.[^41^, ^43^, ^44^] For example, catalysis, organic synthesis, electrochemistry, and biomass processing.^[45]^ In 2019, DESs were applied in the area of spent Li‐ion batteries recycling. The research mainly concentrated on isolating active materials from current collectors and extracting metal ions in positive electrode materials.[^46^, ^47^, ^48^] There is no doubt that the leaching rate and velocity of transition metal oxides (including Co, Ni, Mn, etc.) are typically determined by the acidity, coordination, and reducibility of the leaching solution.

## 2 The Effect of Organic Acid‐Based DESs

In DESs, proton acceptors are the hydrogen ions, and their interaction with oxygen causes water to form, which subsequently triggers the dissolution of the metal oxides around them. Therefore, acidic DESs can effectively dissolve most metal oxides.^[49]^ Currently, DESs with organic acids as hydrogen bond donor components are mainly used as leaching solutions. Organic acids commonly used include FA, acetic acid, MAL, PTSA, OA, citric acid and LA etc..[^50^, ^51^, ^52^, ^53^, ^54^, ^55^, ^56^, ^57^]

### 2.1 The Effect of Binary Organic Acid‐Based DESs

**Figure 5:**
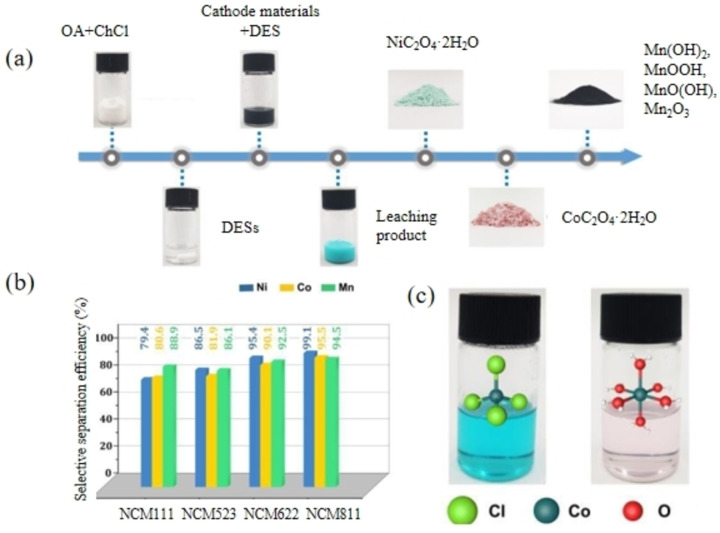
Selective separation of Ni, Co and Mn from spent NCM.^[60]^

Morina et al.^[53]^ found a DES consisting of ChCl and LA with a molar ratio of 2 : 1 leached out nearly 100 % of lithium, cobalt and nickel under conditions of 105 °C for 24 hours. Additionally, this DES can be applied to various positive electrode materials, and effectively reused for three consecutive leaching cycles with a leaching rate of over 80 %. During this process, Ni and Co co‐precipitate and require additional H_3_PO_4_ solution for Li recovery. Lu et al.^[54]^ explored the extraction of Li and Co from spent Li‐ion batteries using various organic acid‐based DESs. MAL and PTSA individually showed good leaching efficiency when synthesized with ChCl, and the efficiency was further improved when they were combined into a DES. Prasetyo et al.^[58]^ also found AA and tannic acid were combined with ChCl to leach out positive electrode metal materials from spent LiCoO_2_ (LCO) powder. The research indicated that tannic acid acted as a strong reducing agent that promoted co‐dissolution through the reduction of metal ions, while AA served as a pH adjuster. The concentration of AA was able to buffer the pH in the acidic range, keeping the metals in the aqueous phase to prevent surface passivation and improve metal solubility, especially Co. Chen et al.^[51]^ discovered that DESs incorporating organic acids as melt components have high acidity, enabling them to dissolve metal oxides. They used FA and its derivatives as leaching agents and designed a series of sequential leaching systems. Under conditions of 90 °C and 12 hours, the ChCl/FA DES achieved leaching rates of 99.8 % for Li and 99.1 % for Co. Building on this, Liu et al.^[59]^ also used ChCl/FA DES at the optimal leaching conditions of 70 °C, 10 minutes, a liquid‐to‐solid ratio (L/S) of 50 g/g, and a water content of 10 % to leach and recover approximately 100 % of Li and Co from LCO. To further validate the influence of different organic acid acidity, researchers used ChCl/OA type DESs for efficient recovery of LCO, with the solvent being easy to recover. The carboxylic acid group in OA, as a hydrogen bond donor, exhibited stronger activity, accelerating the dissolution process. Chang et al.^[60]^ used OAD/ChCl DES with a molar ratio of 1 : 1 to recover spent NCM cathodes. Utilizing a selective dissolution strategy, Ni, Co, and Mn achieved excellent recycling rates of 99.10 %, 95.10 %, and 95.30 %, respectively, within 10 hours at 120 °C (as illustrated in Figure 5(a)). The DES was effective for NCM materials of different formulations, with NCM 811 exhibiting the highest recycling efficiency (as depicted in Figure 5(b)). As a result of the rapid development and commercialization of high‐nickel ternary NCM materials, achieving high recycling efficiency for NCM 811 is particularly appealing. Furthermore, by incorporating dimethyl sulfoxide (DMSO) and water as solvents, the leaching kinetics and mechanism were analyzed in depth. Transition metal coordination was found to be the key to selective dissolution, as indicated by the varying colors in the solution, which reflect different forms of metal ion coordination (as shown in Figure 5(c)). As previously mentioned, achieving selective recycling of metals in batteries requires a longer process and high‐cost DMSO. Thompson et al.^[56]^ found that after leaching with ChCl/OAD DES (molar ratio 1 : 1), Co and Mn were able to co‐precipitate with purified water, easing the recovery technique of spent NCM batteries. Additionally, It has been proven that using a DES consisting of OA and ChCl in a 1 : 1 molar ratio achieves good leaching results for Li and Co from LCO, and this OA‐based DES can be employed repeatedly in various leaching processes with high leaching rates.^[23]^ Xu et al.^[61]^ leached approximately 96 % of Li and Mn from LMO using a ChCl/OA DES at 15 minutes, 75 °C, and S/L 1 g/50 mL. Ma et al.^[62]^ used microwave‐assisted ChCl/OA/H_2_O DES systems and optimized conditions at 100 °C and 10 minutes to selectively leach 99.05 % of Li from LCO and precipitate 99.21 % of cobalt oxalate. Additionally, Roldan et al.^[63]^ used DESs composed of toluene sulfonylamide and ChCl as leaching agents. Due to the strong acidity of toluene sulfonylamide containing sulfonic acid groups, Li and Co leaching rates in lithium cobaltate reached 100 % without the use of any reducing agent, offering advantages such as low operating temperature, short reaction time, and high leaching rates. In terms of developing and screening mild leaching conditions for DES experimental research, it has been found that toluenesulfonic acid‐based DESs can recover approximately 100 % of Li and Co from Spent Li‐ion batteries at temperatures as low as 90 °C within 15 minutes.[^64^, ^65^, ^66^, ^67^] However, toluenesulfonic acid is a highly corrosive acid that can corrode equipment and generate acidic wastewater. DESs composed of ChCl and LA can also leach Li, Mn, Ni, and Co under milder conditions (105 °C, 5 hours).^[68]^ Hydrophobic DESs (decanoic acid and lidocaine) achieve maximum leaching efficiency within 5 seconds under high cobalt concentrations and low liquid‐to‐solid ratios, while sodium oxalate solution can effectively regenerate the DES.^[67]^

In summary, Organic acid‐composed DESs display greater metal dissolution capability under milder conditions. However, it is challenging to selectively recover target metals, and the purity of the co‐precipitates cannot be ensured, becuase of the complex coordination environment created by various acid radical ions. Only a few acids, such as FA^[51]^ and OA,^[53]^ have achieved selective separation of lithium. Additionally, the high molecular weight characteristic of organic acids often results in them having high viscosity. Therfore, compared to polyol‐based DESs, their filtration speed is slower and reaction time is longer.

### 2.2 The Effect of Organic Acid‐Alcohol Based Ternary DESs

In order to improve the leaching efficiency of valuable metals from spent Li batteries, researchers have proposed the addition of alcohol substances to the existing organic acids to form ternary DESs.

**Figure 6:**
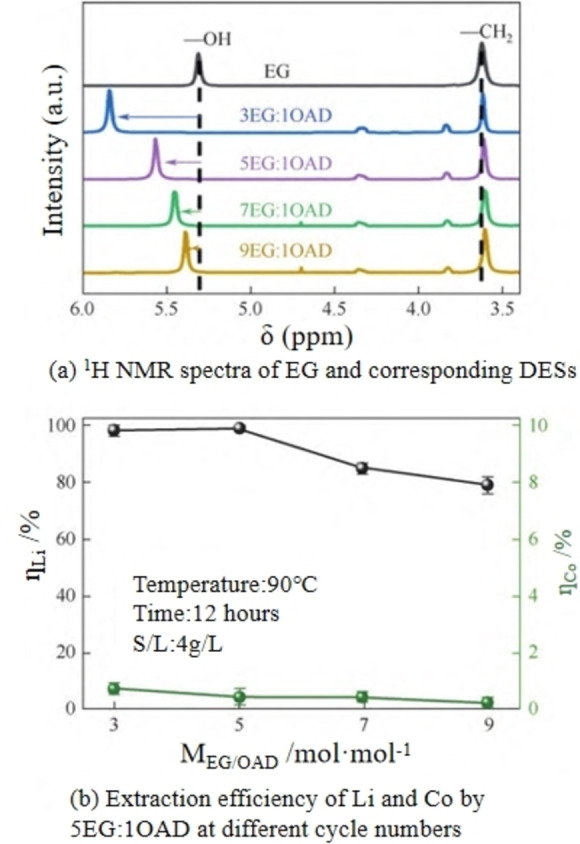
1H NMR spectra of EG and corresponding DESs, as well as the leaching efficiency of 5EG: 1OA for Li and Co at different cycle times.^[69]^

As observed in the leaching experiments of ChCl/EG/organic acid DES systems, the addition of alcohol in organic acid DESs can greatly shorten the leaching time.^[55]^ Tang et al.^[69]^ presented a kind of dual‐functional DES with the ratio of ChC, OAD and EG 1 : 1 : 5 for the selective leaching of metals from spent Li‐ion batteries. Through the examination of the influence of the molar ratio of EG to OAD, an increase in the amount of OAD was found to cause a downward shift in the chemical shift (δ) of the ‐OH group within the prepared DES. This suggests the formation of additional intermolecular hydrogen bonds between the −OH from EG and the −COOH from OAD (as shown in Figure 6(a)). Additionally, the results indicate that increasing the amount of EG within the synthesized DESs enhances system stability and reduces viscosity, whereas an increased acid concentration promotes a complete leaching reaction. To achieve an optimal balance, a 5EG : 1OAD ratio, with a viscosity of 23.5×10^−3^ Pa s, was identified as the most effective, resulting in a lithium leaching rate of 98.2 % after being leached at 90 °C for 12 hours (as shown in Figure 6(b)). Nearly all the lithium was extracted into the leachate, whereas cobalt was found in the precipitate as CoC_2_O_4_ ⋅ 2H_2_O. The results demonstrated that the selective extraction of lithium and cobalt from LCO can smoothly achieved. Furthermore, after the separation of CoC_2_O_4_ ⋅ 2H_2_O, the DES proved reusable for metal leaching and preserved its high leaching effectiveness, reaching nearly 100 %, throughout the next three cycles.

**Figure 7:**
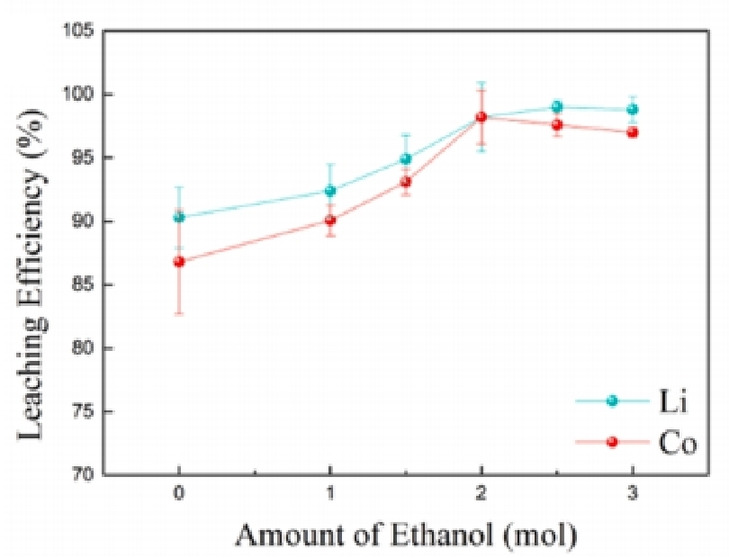
Influence of amount of ethanol added on the leaching efficiency of Li and Co from LCO using ChCl/BSA/ethanol DES at 90 °C, S/L 20 g/L and 12 hours of leaching time.^[72]^

Additionally, Tang et al.^[70]^ constructed a DES consisting of sulfonic acid salicylic acid dihydrate (SAD) and EG. Under operating at lower viscosity, This DES showcased exceptional leaching capabilities, successfully extracting 93.5 % cobalt and 98.3 % lithium from pure LCO. Under the optimal situation of 110 °C and 8 hours, the leaching efficiencies of Li, Co, Ni and Mn from spent LNCM were 100 %, 94.8 %, 99.1 % and 100 %, respectively. Li et al.^[71]^ presented the leaching of cathode active metals in LCO employing a ChCl/EG/BA DES. Under reaction conditions of 200 °C and 2 hours, with a S/L 1/50 mL, the DES system turned deep blue after the reaction, and the leaching rate of Co achieved 96.2 %, showing good fluidity. Li et al. also found the benefits of adding EG, and under optimal conditions of 150 °C, 10 hours and 60 g/L(S/L), the MA : EG DES achieved leaching efficiencies of Li and Co 98.4 % and 98.3 % from LCO. Liao et al.^[72]^ also studied the leaching ability of the ChCl/BSA/Ethanol DES and found that the leaching process was significantly influenced by the quantity of ethanol incorporated into the DES, the DES viscosity effectively was declined as the ethanol content increased. Figure 7 showed the effect of ethanol addition on the leaching efficiency under conditions of 90 °C and a S/L of 20 g/L for 2 hours. Without the addition of ethanol, the leaching rate of Li and Co only achieved 90.3 % and 86.8 %. However, the leaching efficiencies of Li and Co markedly enhanced to 98.2 % as the ethanol rose from 0 to 2 mol. Gong et al.^[73]^ also found that increasing the length of the carboxylic acid alkyl chain would decrease the leaching ability of the DES, and adding water to the DES had a certain influence on the leaching efficiency. Therefore, the strength of acidity alone cannot be the sole criterion for determining the ability to dissolve metal oxides. Additionally, ethanol can also affect the structure of hydrogen bonding in the DES. Due to the reducing nature of ethanol, it promotes the creation of esters between the proton in ChCl and the carbon of the carboxylic acid, thereby enhancing the leaching efficiency. It is evident that the reducing nature of ethanol is also one of the important factors affecting the leaching of positive electrode metal materials.

## 3 The Effect of DESs Reducing Ability

Due to the hydrogen bond donor propylene glycol being a good reducing agent, neutral ChCl/propylene glycol or ChCl/EG also serve as effective leaching agents.

### 3.1 Reducing Effect of Binary Alcohol‐Based DES

EG is widely utilized because it is inexpensive and has a low viscosity. In 2019, Tran et al.^[74]^ first employed ChCl/EG (molar ratio 1 : 2) DES as a leaching agent for LCO recovery and the leaching performance of Li and Co obtained close to 89.8 % and 50.43 %. The leaching effect was not ideal. Therefore, Tran et al. further utilized DES of ChCl/EG and extended the leaching time to extract Co from LCO and NMC. After leaching under 180 °C for 24 hours, the leaching rate of Co reached 99.3 %. Furthermore, Na_2_CO_3_ was added to convert Co^2+^ into CoCO_3_ through chemical precipitation, and the filter residue was then calcined to obtain relatively pure Co_3_O_4_. Additionally, electrochemical deposition can be used to convert Co^2+^ in the leaching solution into relatively pure Co(OH)_2_, allowing the DES to be reused. Furthermore, Tran et al.^[75]^ conducted a approach to recycle spent lithium‐ion batteries using ChCl/EG DES. They found that under the optimal situations of 250 °C and 72 hours, the leaching efficiency of cobalt and lithium was both ≥90 %. This DES does not need the addition of extra reducing agents during the leaching of spent battery cathode materials, and it does not emit harmful gases. However, it requires a higher temperature and longer time, which is not favorable for industrial utilization.

For ChCl/EG DES, EG can reduce lithium cobaltate (LiCoO_2_) to [CoCl4]^2−^ while oxidizing itself to acetaldehyde or acetic acid.^[76]^ These oxidation products can act as complexing agents to form complexes with metal ions, reducing the depletion of metal ions. Clearly, in alcohol‐based DESs, the leaching mechanism is different from carboxylic acid‐based DESs, and the reducing properties of alcohol‐based DESs play a crucial part in the extraction process of Li‐ion battery cathode materials. Compared to carboxylic acid‐based DESs, alcohol‐based DESs are more cost‐effective. Additionally, Tran et al.^[77]^ found that EG can not only reduce Co^3+^ to Co^2+^ in LCO, but also reduce high‐valence Mn to low‐valence in ternary lithium batteries. Without the addition of reducing agents, acids or bases, lithium cobaltate can be dissolved in ChCl/glycerol DES at 80 °C, achieving a high leaching rate of 94.1 % for Co element,^[47]^ comparable to or even better than traditional wet dissolution efficiency. Wang et al.^[78]^ presented that the DES (ChCl/EG) was able to recyle metals from NCM 111. Under optimal experimental conditions of 180 °C and 24 hours, the leaching rates of Li, Co, Ni, and Mn reached ≥91 %. Co, Ni and Mn can be co‐precipitated using H_2_SO_4_ and NaOH solutions to compensate for their losses and achieve a 1 : 1 : 1 molar ratio for the regeneration of the electrode with excellent electrochemical performance. Schiavi et al.^[76]^ also used the same 1ChCl/2EG to recycle Co from spent electrode powder (black powder) containing various cathode materials, Al, Cu and Fe impurities. In spite of the powder contains abundant metals, this DESs can selectively recover Co with an extraction rate as high as 90 % (Ni only 10 %), while copper and aluminum can be extracted in the pretreatment process, bringing additional profits. However, the solvent metallurgical recovery route is cumbersome for each element, requiring extractants such as D2EHPA and OA solution.

**Figure 8:**
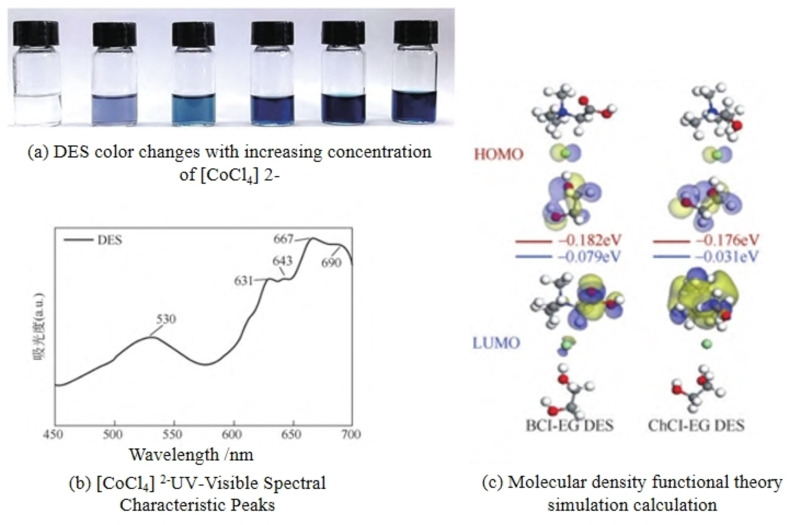
Solubilization ability of DES.^[80]^

In addition, Polyethylene glycol (PEG), due to its advantages of biocompatibility, non‐corrosiveness, and environmental friendliness, is also an excellent hydrogen bond donor. Chen et al. developed a kind of PEG/ChCl DES with a molar ratio of 2 : 1, achieving great solubility of LCO under 80 °C and 24 hours.^[79]^ Luo et al.^[80]^ used betaine hydrochloride (BCl) and EG to prepare DES for leaching Li, Ni, Co, and Mn from spent NCM without a reducing agent. The leaching efficiencies of all valuable metals reached 99 %. Over the DES leaching process, EG reduced the metal ions, and Cl‐ formed [CoCl4]^2−^ to promote the leaching of NCM. This was validated by the color change of the leaching solution (the concentration of [CoCl4]^2−^ increased, resulting in a transition from transparent to blue, as shown in Figure 8(a)), analysis of the Ultraviolet (UV) visible spectra of the filtrate (characteristic peaks at 690 nm, 667 nm, 643 nm and 631 nm indicated the presence of [CoCl4]^2−^, as shown in Figure 8(b)), and computational analysis using density functional theory (EHOMO values reflecting the electron‐donating ability of the molecules, EHOMO‐(BCl‐DES)>EHOMO(ChCl‐DES), as shown in Figure 7(c)). These findings indicated that BCl‐DES had a stronger solubilization ability due to its easier binding with vacant orbitals of transition metal ions.

The reduction of transition metals in the leaching process of Li‐ion battery cathode materials using DESs is typically controlled by hydrogen bond donors, which reduce Co^3+^ to form metal complexes [CoCl4]^2−^ that are soluble in the DES system.[^81^, ^82^, ^83^] Therefore, numerous researchers have developed and screened DES with strong reducibility and mild leaching conditions. Ethanol contributes to the leaching system possibly due to its reducing capability, which helps to stabilize the structure of Co^2+^ by reducing Co^3+^. Kinetic studies have shown that ethanol not only acts as a mass diffusion accelerator but also functions as a reducing agent. In recent years, new green reducing agents have played a criticl role in the acid leaching of metals from cathode materials. Ethanol has been used as a reducing agent in H_2_SO_4_ to leach LCO, resulting in the leaching of over 99 % of Co and Li.^[84]^ By oxidizing ethanol to acetic acid, Co^3+^ is reduced to Co^2+^. Zhao et al.^[85]^ introduced ethanol as a reducing agent in the H_2_SO_4_ leaching of valuable metals Li and Co from cathode materials and found that the influence of ethanol as a reducing agent is similar to that of hydrogen peroxide (H_2_O_2_), ultimately achieving leaching rates of around 99 % for both Co and Li.

Polyol‐based DESs, being the earliest DESs employed in leaching metallic materials, have garnered praise owing to their low viscosity, low reagent cost, biodegradability, and capacity to directly handle cathode materials. However, the demanding operational conditions of >180 °C and 12 h remain an obstacle to the further application of lithium‐ion batteries recycling. Therefore, DESs with higher activity are gaining more attention

### 3.2 Reductive Effects of H_2_O_2_ and O_3_

H_2_O_2_ is a strong oxidizing agent that exhibits significant reducing properties in chemical reactions. When hydrogen peroxide comes into contact with metal oxides or sulfides in spent batteries, a redox reaction can occur, converting these insoluble metal compounds into soluble metal salts. Additionally, hydrogen peroxide can break down the passivation layer in batteries, exposing more metal surfaces and improving the efficiency of the leaching reaction. Lee et al.^[86]^ reported the variation in leaching rates of metallic elements in cathode materials when a small amount of H2O2 was introduced to act as a reductant. After adding a small amount of the reductant, the leaching efficiencies of Li and Co both ≥95 %, indicating a significant improvement in leaching rates with the introduction of H2O2 as a reductant.

Meanwhile, researchers have also studied the selective in‐situ leaching of Li from spent lithium‐ion batteries cathode active material (LiFePO_4_) using green DESs (ChCl/EG) under an ozone (O_3_) atmosphere.^[87]^ The findings indicated that the presence of moisture in the O3 atmosphere greatly promoted the leaching of Li. Under the optimal conditions of 40 °C, 6 hours, S/L 20 g/L and 8EG : 1CC, Li was leached over 92.2 %. This is because spent Li‐ion batteries are mainly composed of cathode materials, anode materials, electrolytes, and separators. The cathode materials typically contain metals including Li, Co and Ni, while the anode materials mainly consist of carbon, silicon, and other elements. While the leaching process is ongoing, incorporating water as a reductant can promote redox reactions between metals and water. The hydrogen element in water molecules has some reducing properties and can undergo redox reactions with metal oxides in the battery, reducing them to metallic ions and consequently improving the leaching efficiencies of the metal elements. Water molecules can also facilitate the reaction between the leaching agent and metal elements. They can form hydrated ions with the leaching agent, thereby increasing the contact area and reactivity between the leaching agent and metal elements, further promoting the dissolution of the metal elements.

### 3.3 Reductive Effects of Cu and Al In Current Collectors

In industrial waste of discarded spent lithium‐ion batteries, there are generally other metal impurities present, such as copper and aluminum foils used as current collectors. The aforementioned studies did not consider the leaching of impurities like copper in the current collector. In addition to the reducing properties of DES itself, the current collectors of spent Li‐ion batteries can also act as reducing agent to improve the leaching of transition metals. Joulie et al.^[88]^ also pointed out that Al or Cu can reduce Co^3+^, but did not elaborate on the combined leaching effect of these two metals in lithium‐ion batteries. And Peng et al.^[89]^ presented the combined leaching infulence of Al and Cu. However, the aforementioned studies were based on traditional acid leaching methods.[^88^, ^89^]

Peeters et al.^[90]^ found that compared to ChCl, CA, and HCl solutions, ChCl/CA with a molar ratio of 1 : 1 and added 30 wt % H_2_O exhibited stronger dissolution capacity for elemental Cu, Al, and LCO. The possible reason is that Cu plays a role as a reducing agent in this process, where Cl^−^ disrupts the crystal structure of LCO and Cu reduces Co^3+^ to Co^2+^, forming Cu^2+^ and Cu^+^ while complexing with Cl^−^ to form [CuCl_2_]^−^. Compared to this DES system, in PEG/CA (1 : 1), the ‐OH of CA reduces Co^3+^ to Co^2+^, consistent with the observation of a pink color in the leaching solution. The ‐O‐ on the long chain of PEG200 in DES combines with electron‐deficient species Li^+^ and Co^2+^, thereby promoting leaching.^[91]^ At lower water content, CA is less ionized, resulting in a lower leaching rate for Cu. Based on this, Peeters et al.^[90]^ further used ChCl/CA DES to leach LCO powder containing copper and aluminum metals. The study showed that when using this DES to directly leach the positive electrode and current collector mixed materials, no additional reducing agents are needed. Under optimized conditions (40 °C, 60 min, 1 g/20 mL), they leached out 98 % of Co^2+^, 38 % of Al^3+^, and 94 % of Cu^2+^/Cu^+^. The metallic copper and aluminum themselves function as reductants and facilitate the leaching of Co and Li. This study provides a method for directly leaching LCO without removing the aluminum foil and copper foil. However, due to the rapid dissolution of Cu and Al, the subsequent separation and purification steps for metal ions become more complex and require a large amount of extractant. Although direct leaching of the positive electrode and current collector mixed materials using DES has advantages such as mild operating conditions, no need for additional reducing agents, and less release of harmful gases, it is still limited by the selectivity for the positive electrode material. Considering cost, Yu et al.^[92]^ proposed a leaching process using Cu and Al foils from discarded Li‐ion batteries as reducing agents, achieving leaching rates close to 99.9 % for Co, Li, and Ni.

To further highlight the impact of reducing properties on the leaching of positive electrode metal materials, researchers conducted cyclic voltammetry experiments on the DES and the DES after leaching LCO.^[93]^ The cyclic voltammogram of the DES after leaching LCO showed a highly similar to that of CoCl_2_.^[94]^ This experiment demonstrated that Co in LCO had undergone a conversion from Co^3+^ to Co^2+^ during the dissolution process in the DES, confirming the effect of reducing properties on the dissolution of LCO in DES.[^90^, ^95^] By selecting appropriate reducing agents and controlling their dosage, the leaching process can be optimized to improve metal dissolution rate and selectivity.

## 4 Influence of Coordination

DES is a low‐melting mixture composed of hydrogen bond acceptors and coordinators (such as metal salts, metal complexes, or hydrogen bond donors) in certain stoichiometric ratios.[^96^, ^97^] Tran et al.^[75]^ contrasted the leaching influences of LCO and NCM using ChCl/EG DES (molar ratio 1 : 2) as a leaching agent, and found that the more types of metal oxides present, the more complex the process required to recover the valuable metals. Zhang et al.^[98]^ leached out valuable metals using ChCl/acetic acid and ChCl/MA, and then synthesized NCM materials using the sol‐gel method. It was observed that NCM‐ChCl/MA had superior properties compared to NCM‐ChCl/AC. This is because in the sol–gel approach, the two acids have different coordination abilities. Maleic acid esterification forms a stable network that chelates metal ions, while acetic acid's weak chelation ability can lead to impurity formation. Therefore, under the same solid‐liquid ratio, a large number of valuable metal ions need to compete for a limited number of binding sites in the solution. Ternary lithium batteries contain more valuable metal ions, thus requiring more binding sites.

Zeng et al.^[99]^ compared the leaching influences of ternary DES and binary DES with different water contents on lithium cobalt oxide‐copper mixed powder. The experiments investigated the leaching effects of different DESs on LCO‐copper mixed powder at 100 °C, 1/100 g/g, and 5 hours. The results showed that the PEG‐CA (1 : 1) DES had a low Cu leaching rate of 2 %, while the leaching efficiencies for Li and Co were 96 % and 71 %, respectively. However, when 10 wt % deionized water was added to the PEG‐CA (1 : 1) DES, the Cu leaching rate significantly increased to 24 %. Although the addition of a small amount of H_2_O effectively reduced the DES viscosity, the ternary DES did not significantly improve the leaching performance of Li and Co. This is because during hydration, the structure of the DES is slightly disrupted, reducing the coordination number between molecules to 90 % of pure DES.^[100]^ The water molecules in the DES act as secondary hydrogen bond donor molecules and do not have a significant leaching effect on CO and Li.^[101]^ Li et al.,^[39]^ in their reseach on the leaching of LCO in the ChCl/EG/BA DES system, analyzed the leaching solution and residue. In order to verify the establishment of intermolecular hydrogen bonds between the hydrogen bond donor and acceptor, FT‐IR spectra of both the pure components and the prepared DES were provided by them. The prepared DES contained the characteristic peaks of the functional groups associated with ChCl, EG, and BA.[^102^, ^103^, ^104^, ^105^, ^106^] The FT‐IR spectra of EG, BA, and DES were employed to explain the shifts in the characteristic peaks of the functional groups after DES synthesis. The stretching vibration band of the CCO functional group of ChCl occurred around 956 cm‐1, indicating that the Ch^+^ group did not change in the prepared DES. The peak shape of the O−H stretching vibration broadened, and the peak position hardly changed (3296.3 cm^−1^), indicating the formation of many intermolecular or small intramolecular hydrogen bonds. The characteristic peaks of the C−O stretching vibration (EG) appeared at 1083.0 and 1037.0 cm‐1, shifted from 1078.0 and 1033.2 cm‐1, respectively. The characteristic peak of the −C=O stretching vibration in BA showed a shift from 1683.3 cm^−1^ to 1708.1 cm^−1^. The stretching vibration characteristic peak of the −C=O group in the prepared DES exhibited a slight shift from 1078.0 cm^−1^ to 1712.1 cm^−1^. The higher energy indicates a change in the electronic environment of the −C=O group, possibly accompanied by the generation of metal ions with oxygen coordination. DES composed of hydrogen bond donors and ChCl (hydrogen bond acceptor) forms hydrogen bonds between the hydrogen bond donor and chloride ions, leading to the dissolution of metal ions.^[107]^ In the current system, whether multiple strong coordination environments exist to accelerate cobalt dissolution and related dissolution mechanisms requires further study.

Choosing suitable coordinators can reduce the activation energy of metal ions in the solvent, thereby improving the leaching rate, achieving selective leaching of specific metal ions, avoiding interference from other impurities, and facilitating the desorption of metal ions from the solid surface into the solvent, thereby improving the leaching efficiency. Coordination has a significant impact on the leaching of positive electrode metal materials in DESs. By selecting appropriate coordinators, the leaching rate can be improved, selective leaching can be achieved, and the leaching efficiency can be enhanced. Therefore, in the recycling process of positive electrode metal materials from waste lithium‐ion batteries, the optimization of leaching performance of low‐melting solvents can be considered by utilizing coordination effects. Future research can further explore the types and concentrations of coordinators and their synergistic effects with other leaching conditions to achieve efficient and environmentally friendly recovery of positive electrode metal materials from waste lithium‐ion batteries.

## 5 Conclusion and Outlook

Table 1 summarizes the composition and leaching efficiency of some DESs. In acidic DES leaching systems, most of the organic acids involved have certain reduction properties, and they not only act as leaching agents but also serve as reducing agents.^[108]^ However, in neutral or alkaline DES leaching systems, the role of hydrogen ions is significantly weakened, and the dissolution of metal oxides mainly relies on the coordinating ability and reduction properties of DES components.[^109^, ^110^, ^111^] Regardless of the type of DES system, the leaching mechanism involves the transformation of high‐valence transition metal oxides into low‐valence coordination compounds. For example, Lu et al.^[54]^ proposed that the dissolution of LCO in the ChCl : H2C2O4 DES system involves the replacement of Li in LCO by H^+^ from H2 C2O4, leading to the destruction of the LCO crystal structure.^[50]^ Co^3+^ is then reduced by the reductive C_2_O_4_^2−^ to Co^2+^, which subsequently forms [CoCl_4_]^2−^ by coordination with Cl^−^. Tang et al.^[69]^ described the leaching mechanism of cobalt and lithium in LCO in DES and the corresponding reaction equations,[^50^, ^69^] including the replacement of interlayer Li^+^ in LCO by H^+^ in DES, the reduction of Co^3+^ by EG, and the formation of Co^2+^ complexes with carboxyl/sulfonic acid (SA) groups. UV spectra of the leaching solution also confirmed these results. Zeng et al.^[112]^ used EG/CA to recover ternary lithium‐ion batteries, where EG was oxidized to an aldehyde, acting as a reducing agent to facilitate the conversion of high‐valence elements (Ni, Co, Mn) into Ni^2+^, Co^2+^, and Mn^2+^ ions. This led to the decomposition of LiNi_1/3_Co_1/3_Mn_1/3_O_2_, and the aldehyde promoted the formation of citrates, which inhibited the leaching of aluminum foil. UV spectroscopy and Fourier Transform Infrared Spectroscopy (FTIR) spectra demonstrated the chemical changes during the leaching process. Li et al.^[52]^ proposed that the dissolution reaction at the interface between LCO and ChCl : OA involves the transformation of cobalt elements in the solid lattice into ionic cobalt during leaching, as well as the continuous reduction of Co^3+^ to Co^2+^. The complexation of Co^2+^ compounds has a higher solubility,[^113^, ^114^] mainly due to the active proton and reduction properties of OA, which promote the breakage of original metal oxide bonds and contribute to the improvement of leaching efficiency.

Some researchers believe that metal oxides are strong oxidants in acidic environments, where some Cl^−^ is oxidized to Cl_2_, and some Cl^−^ participates in coordination with Co^2+^ to form coordination ions [CoCl_4_]^2−^, indicating that Cl^−^ rather than acid ions acts as a reducing agent in acidic DES. For example, Tian et al.^[102]^ proposed that during the dissolution of LCO in LA/guanidine hydrochloride, the presence of acidic lactic acid causes LCO to act as a strong oxidant, while Cl− acts as a reducing agent to reduce Co^3+^ to Co^2+^. Cl− ions in hydrochloric guanidine coordinate with Co^2+^ to form [CoCl_4_]^2−^ anions, resulting in dissolution. Chang et al.^[60]^ used thermogravimetric‐mass spectrometry (TG‐MS) to simulate the leaching reaction on‐site and confirmed this viewpoint. During the experiment, the signal intensities of mass‐to‐charge ratios (m/z) 35 and 36 were three times higher than those of 37 and 38, consistent with the natural abundance ratio of chlorine isotopes. Based on the principle of conservation of valence, it was indicated that choline chloride participates in the redox reaction to generate chlorine gas. The signal at m/z=16 indicates that the generation of oxygen lags behind the appearance of chlorine, providing effective evidence for the reaction between chlorine gas and water to produce oxygen gas at high temperatures. However, Nand's team^[115]^ used a series of analytical detection methods and controlled variables during the leaching process of LCO using 1ChCl : 2EG, which refuted the idea that Cl^−^ acts as a reducing agent and is oxidized to Cl_2_ during leaching. This contradicts the results of Chang et al.′s^[60]^ study. Therefore, the verification results regarding whether Cl^−^ acts as a reducing agent in the leaching mechanism still require further investigation.

In conclusion, DESs have shown promising potential for the leaching of metal oxides from various sources. The composition and properties of DESs play a crucial role in their leaching efficiency. Acidic DES systems often involve organic acids with reduction properties, while neutral or alkaline DES systems rely on the coordinating ability and reduction properties of DES components. The leaching mechanisms involve the transformation of high‐valence metal oxides into low‐valence coordination compounds. Further research is needed to explore the leaching mechanisms of different DES systems and validate the role of Cl^−^ as a reducing agent. Additionally, future studies should focus on optimizing DES compositions, exploring new DES formulations, and investigating the synergistic effects of various factors to improve the leaching efficiency of metal oxides from different sources.

Currently, the research on using DESs for leaching cathode active materials is still in its early stages, and there is a lack of systematic studies.^[118]^ In China, the conventional combination of acid leaching and chemical precipitation methods is commonly used for the recycling of spent lithium‐ion batteries. However, the use of corrosive strong acids is not only hazardous but also costly and complex. Additionally, the treatment of waste solutions and the restrictions on toxic gas emissions hinder the development of this technology. Therefore, the development of green and efficient leaching agents and reducing agents remains a major research direction for the future recycling of lithium‐ion batteries. It is necessary to understand the leaching mechanism, kinetics, and thermodynamic properties of cathode materials in DESs. Exploring milder dissolution conditions and improving dissolution efficiency are essential. The study of DES components on the solubility of different metal ions can achieve hierarchical dissolution of different metal ions. Exploring optimal dissolution conditions and efficiency and obtaining effective theoretical and technical support based on organic acid DESs for the leaching and preparation of cathode materials for lithium‐ion batteries will provide effective theoretical and technical support for the recycling and utilization of lithium‐ion batteries in new energy vehicles. The main directions for further research include the following:

1. Screening the optimal DESs and conditions for leaching and separation of metal ions from different cathode materials in different types of batteries. Cathode materials such as lithium iron phosphate, ternary lithium, and lithium cobalt oxide contain various metal ions. Exploring the simple and efficient leaching mechanism and hierarchical leaching of cathode materials in DESs is still a hot topic in future research. Currently, it is challenging to separate metal ions after DES leaching, as metal ions form complexes with DES components and must be achieved through the addition of precipitants or electrodeposition, both of which have drawbacks. Therefore, it is necessary to develop DESs that can directly separate metal ions without damaging the solvent itself.
2. Further research and improvement of the theoretical system for the existence state and leaching kinetics of metal ions in cathode materials. Cathode materials often exhibit element loss after multiple cycles, so it is necessary to use characterization techniques such as inductively coupled plasma atomic emission spectroscopy (ICP) to determine the state and content of metal ions, the types and concentrations of various metal ions, and calculate the leaching rate to determine the amount of replenishment required for subsequent in‐situ preparation. The advantages of the corresponding DESs can be determined by comparing the states of metal ions in inorganic acid and organic acid leachate. Then, the apparent reaction rate and corresponding activation energy can be calculated using the unreacted shrinking core model to determine the leaching control step. In addition, although there have been extensive studies on the dissolution mechanism and kinetics of DESs, the analysis of the evolution of element existence states, enrichment laws of interfacial reactions, and molecular driving forces are still incomplete. Therefore, it is necessary to further improve the theoretical system of DES leaching, design DES leaching agents from a theoretical perspective, and meet the requirements of high selectivity, low viscosity, good recyclability, and stability to further improve leaching efficiency.
3. Research on more efficient pre‐treatment technologies for spent lithium‐ion batteries is also necessary. Current recycling processes for spent lithium‐ion batteries mostly involve mechanical crushing into black powder, which makes the leaching of cathode materials in DESs difficult. This not only reduces the leaching efficiency of DESs but also increases the cost of impurity removal due to the introduction of copper and aluminum ions. Therefore, it is necessary to develop new pre‐treatment technologies for spent lithium‐ion batteries to reduce the introduction of impurities (graphite, copper, aluminum).
4. Further research is needed on process factors that hinder large‐scale production. Existing DES leaching experiments have achieved good separation results on a small scale, but there are still many difficulties in pilot‐scale and large‐scale operations. For example, some DES components are expensive, and some DES reaction conditions have stringent requirements. These issues will affect the scale‐up production. Therefore, it is necessary to further optimize the parameters such as temperature, time, and reactant ratio of DES reactions and conduct cyclic experiments.

In addition, compared with traditional wet solvent processes, the drawback is that after extracting metal ions using deep eutectic solvents, the extraction process may face some challenges. Extractants such as PC‐88A and CYANEX are widely used in traditional processes, but DES may have the following drawbacks in some cases: (a)Selectivity issue: The selectivity of DES may not be as high as that of extractants, which may lead to the simultaneous extraction of other impurities or metal ions during the leaching process, thereby affecting the purity of the final product. (b)Solubility limitation: Some metal ions may have a relatively weak coordination ability with DES, resulting in difficulty in effectively extracting them after leaching. This may require further optimization and adjustment of the extraction conditions to improve the extraction efficiency. (c)Solvent stability: The stability of DES may be affected by factors such as temperature and pH. In practical applications, it is necessary to ensure the stability and compatibility of the solvent to avoid changes during the extraction process. (d)Cost and sustainability: The preparation cost of DES may be relatively high, and in some cases, it may not be easy to recover and recycle. This requires comprehensive consideration of factors such as cost and sustainability.

It should be noted that these drawbacks are not ubiquitous, and can be mitigated or overcome through appropriate experimental design and optimization. In addition, DES may have advantages in other aspects, such as environmental friendliness and low volatility. When selecting leaching and extraction methods, various factors should be taken into account, including the target metal, solution characteristics, cost, and environmental requirements, to determine the most suitable process.

1. DESs for Spent lithium Battery recycling have been reported for the leaching of cathode materials as organic extractants and for the removal of Polyvinylidene fluoride (PVDF) binders. However, DES applications for LiFePO4 (LFP) batteries have not been carried out.^[119]^ Nevertheless, some studies have used LCO and NMC battery leaching^[118]^ and separation to remove Fe(III) from the mixed battery leaching liquor.^[76]^ In this sense, applications in Fe(III) solutions may be analogous to the extraction from LFP leaching solutions. Zhu et al.^[118]^ studied DES for the extraction and separation of Li(I), Co(II), Ni(II), Mn(II), and Fe(III) and subsequent recovery. Aliquat336 and the last environmentally friendly solvent, L‐menthol, were mixed, and the eutectic point was determined. The characteristics of the optimal proportion (3 : 7 Aliquat336/L‐menthol molar ratio) and metal separation extraction curves were determined. Extraction tests were carried out at 25 °C, under atmospheric pressure, and an O/A ratio of 1/5, and the phases were mixed at 35 rpm until equilibrium. The aqueous phase was a leaching HCl solution of mixed metals. The results showed that the recovery rate of Fe(III) was 99 % for all the parameters studied, without Li coextraction. Therefore, HDES based on Aliquat 336/L‐menthol was used as the only extractant for efficient metal separation without using volatile and toxic organic solvents. Schiavi et al.^[76]^ proposed a choline chloride‐ethylene glycol deep eutectic solvent (ChCl : EG) for recovering cobalt from the electrode powder of spent lithium‐ion batteries (LIBs). The electrode powder included Fe impurities from the physical treatment of LCO batteries, and the eutectic solvent was used to recover Co without extracting Fe. The above‐mentioned eutectic solvent was at 90 °C, and after 24 h of leaching, Cu was selective; in addition, a second leaching with the same mixture at 180 °C and 20 h leached Co and Mn, and in both processes, Fe impurities remained at the solid state. Since Fe(III) is a common impurity in LIBs hydrometallurgical processing, this process may separate Fe and increase the purity of other batteries. Although above researches can provide some references for the recovery of valuable metals in LFP cathode materials, the DES leaching and extraction research based on LFP is undoubtedly lacking, especially in China, and more research in this regard should be carried out in the future.
2. Accelerate the development of international standards related to the industry. With the popularity of electric vehicles, the number of spent lithium‐ion batteries will increase exponentially. Countries encourage the recycling of spent lithium‐ion batteries, but there are no clear policies and standards regarding the recycling and pollution emissions of DESs. To accelerate the industrialization process, the formulation of relevant standards needs to be prioritized.

## Nomenclature

DES: Deep eutectic solvent
LCO: LiCoO_2_
NCM: Lithium nickel cobalt manganese oxide
ChCl: Choline chloride
FA: Formic acid
OA: Oxalic acid
OAD: Oxalic acid dihydrate
CA: Citric acid
BA: Benzoic acid
MAL: Malic acid
PTSA: P‐toluenesulfonic acid
LA: Lactic acid
UV: Ultraviolet
FTIR: Fourier transform infrared spectroscopy
ICP: inductively coupled plasma atomic emission spectroscopy
TG‐MS: thermogravimetric‐mass spectrometry

## Conflict of Interests

The authors declare no conflict of interest.

6

## Biographical Information

*Chongyu Li graduated from University of Shanghai for Science and Technology, with a master's degree. He is currently employed at the School of Intelligent Manufacturing, Shazhou Professional Institute of Technology, and holds the position of laboratory director. He has extensive experience in new energy vehicle technology and higher vocational education*.

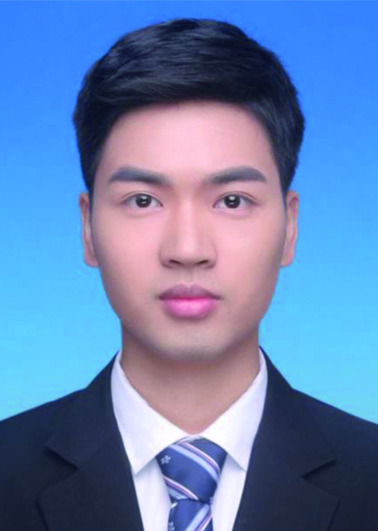

## Biographical Information

*Dr. Jianjiao Jin is employed at Shazhou Professional Institute of Technology. He has extensive teaching and research experience, within‐depth research and understanding in the field of new energy technologies, particularly in the area of power battery recycling*.

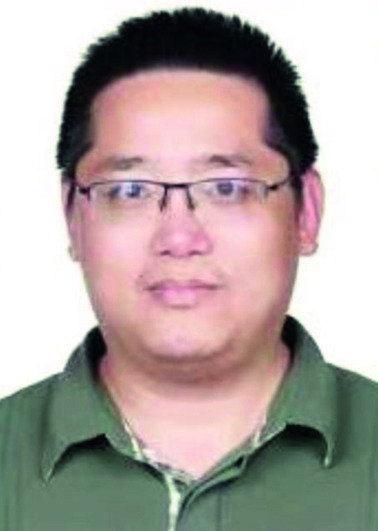

## Biographical Information

*Zhang Yuan is a junior student majoring in New Energy Vehicle Technology at Shazhou Professional Institute of Technology*.

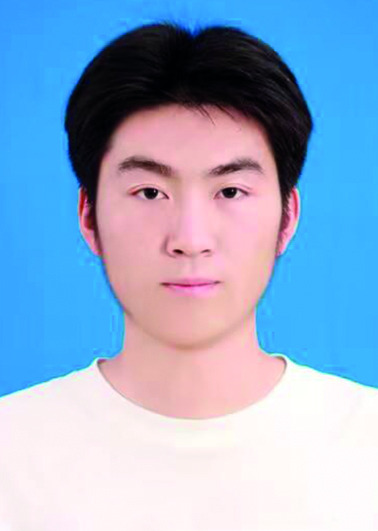

## Biographical Information

*Dr. Chenyun Zhang graduated from the School of Chemistry and Chemical Engineering at Shandong University and is currently employed at Wuxi Vocational Institute of Arts & Technology. Her research expertise lies in the preparation of water splitting catalysts using ionic liquids or deep eutectic solvents*.

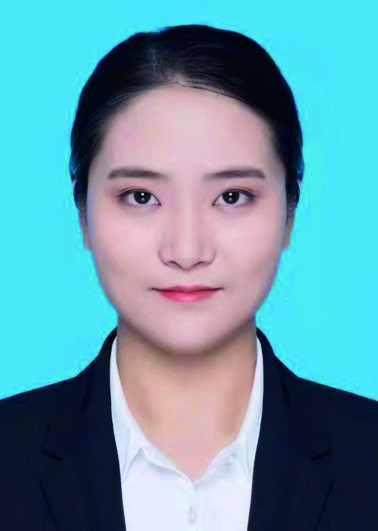

## Biographical Information

*Associate Professor Liangqin Wu is the director of the Automobile Service and Engineering Teaching and Research Office of Shazhou Professional Institute of Technology. he is an education expert with over 20 years of rich experience in higher vocational education. he has been in charge of the construction of several majors in the school and undertaken many provincial‐level scientific research projects in Jiangsu Province (China)*.

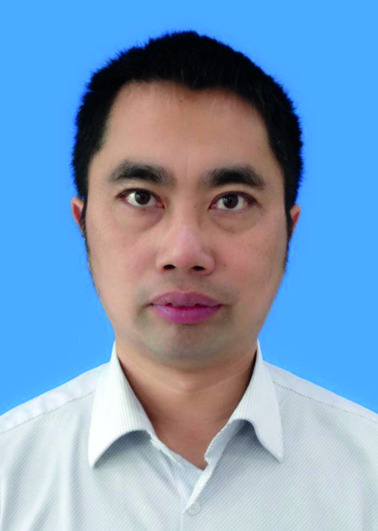

## Biographical Information

*Chu Wang is a sophomore student majoring in New Energy Vehicle Technology at Shazhou Professional Institute of Technology*.

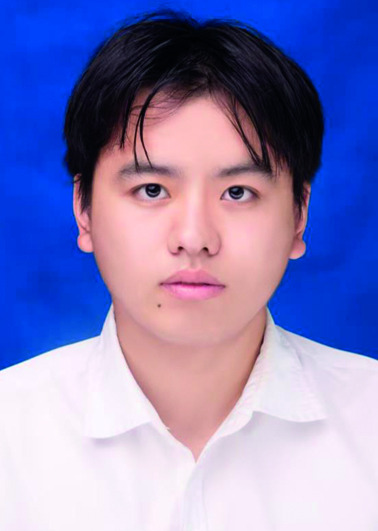

## Data Availability

The data that support the findings of this study are available on request from the corresponding author. The data are not publicly available due to privacy or ethical restrictions.
